# Impact of social-psychological factors on low-carbon travel intention: Merging theory of planned behavior and value-belief-norm theory

**DOI:** 10.1016/j.heliyon.2024.e28161

**Published:** 2024-03-21

**Authors:** Yan He, Yilin Sun, Zhijian Zhao, Mengwei Chen, E. Owen D. Waygood, Yang Shu

**Affiliations:** a Institute of Intelligent Transportation Systems, College of Civil Engineering and Architecture, Zhejiang University, Hangzhou, 310058, China; b Center for Balanced Architecture, Zhejiang University, Hangzhou, 310058, China; c The Architecture Design & Research Institute of Zhejiang University Co., Ltd., Hangzhou, 310013, China; d Polytechnic Institute & Institute of Intelligent Transportation Systems, Zhejiang University, Hangzhou, 310058, China; e School of Design and Architecture Zhejiang University of Technology, 288 Liuhe Road, 310023, Hangzhou, China; f Department of Civil, Geological, and Mining Engineering, Polytechnique Montréal, 2500, Chemin, de Polytechnique Montréal, Canada

**Keywords:** Low-carbon travel, Incentive, Theory of planned behavior, Value-belief-norm theory, Structural equation model

## Abstract

Low-carbon travel assumes paramount significance in energy conservation and the establishment of an eco-friendly transportation ecosystem. This paper endeavors to explore the relationship between low-carbon travel intention, latent psychological variables, and sociodemographic attributes, drawing insights from responses of 602 residents in Hangzhou, China by structural equation model and multi-group model. In particular, we synthesize the theory of planned behavior, value-belief-norm theory, and view of incentive, a reflection of the public support for incentive policies. Results reveals that the primary determinants influencing the low-carbon travel intention encompass the view of incentive, attitude, and subjective norms. Individuals with diverse sociodemographic attributes manifest varying sensitivities, with males and elders exhibiting heightened responsiveness to incentive, while the presence of children decrease the attraction of incentive. These findings demonstrate that low-carbon travel intention can be increased by three ways, one is by the strong attraction of incentive especially tailor incentive policy, another is by making family-friendly policies to facilitate travel for groups with children, and the last is by improving the quality of low-carbon travel services thus increasing the attitude and other determinants.

## 1 Introduction

In recent years, the conspicuous manifestations of climate change stemming from the greenhouse effect have escalated, endowing human existence with profound implications. At the epicenter of this environmental challenge lies carbon dioxide emissions, the principal culprit behind the greenhouse effect, compelling the Chinese government to set forth aspirations of achieving carbon emission peaking by 2030 and carbon neutrality by 2060. However, empirical inquiries have elucidated that transportation-related carbon emissions substantiate a significant 14% of the nation's carbon footprint [1], how to reduce traffic carbon emissions has become a major challenge. One of pro-environmental behavior, low-carbon travel, which, as the name suggests, refers to the use of green and energy-efficient modes, is an important means of addressing this challenge. Thus, scholars have focus on unravelling the mechanism underlying low-carbon travel behavior to reduce carbon emissions.

Behavioral intention serves as a pivotal determinant of behavioral decisions [2], implying that individuals' implementation of low-carbon travel behavior is closely connected with their low-carbon travel intention. Thus, the establishment of a conceptual framework for low-carbon travel intention assumes paramount importance, enabling an incisive analysis of low-carbon travel behavior. In the embryonic stage of pro-environmental behavior studies, models predominantly revolved around attitudes, pro-environmental knowledge, etc. [3]. Subsequently, the theory of planned behavior (TPB) was applied to encompass investigations in pro-environmental domains, ranging from waste classification behavior to carbon offsetting behavior [4,5]. Meanwhile, other scholars explored pro-environmental behavior through the perspective of the value-belief-norm theory (VBN) [6,7]. Researchers have validated the applicability of TPB and VBN in the study of low-carbon travel behavior, latent variables like low-carbon awareness are then incorporated into models, giving rise to a series of extended TPB or VBN models [8,9]. The advent of these extended models has brought novel insights to researchers. Bamberg and Möser [10] underscored the combination of these two theories enhances the interpretability of behavioral intention modeling analysis. This point finds affirmation through the works of Han [11] and Klöckner [12], who employed a similar joint modeling approach. However, it is imperative to acknowledge that such models remain limited to the psychosocial perspective of individuals, overlooking the potential impact of policy implementation on behavioral intention. As we all know, policy, as a macro-control tool, works by guiding people's behavior and plays an important role in social development. Exploring the effects of policy implementation helps to analyze the shortcomings of policies, provides guidance for formulating, evaluating and optimizing policies, and provides targeted recommendations for solving relevant problems simultaneously.

The government has instituted a plethora of travel demand management policies with the aim of alleviating the growth of private car ownership, prioritizing public transportation, and guiding travelers towards adopting low-carbon travel modes, such as license plate rules on travel restriction which has been shown to have a significant impact on promoting low-carbon travel [13]. Furthermore, the Chinese government has set a commendable objective for major cities to achieve a public transportation travel proportion of 40% or higher [14]. Nonetheless, the current share of low-carbon travel is still low compared to developed countries in the Asian region, such as Singapore and Japan [15]. In light of this circumstance, a novel incentive mechanism known as the “low carbon behavior reward system” (ILBRS) has been proposed to propel the cause of low-carbon development [16]. This system is based on the premise of a sound carbon trading market, in which usually use models such as shapley model to rationally distribute carbon emission reduction revenues among enterprises [17], so as to achieve cross-enterprise flow of carbon emission reduction resources, quantify carbon emission reduction behaviors and reward energy-saving behaviors. As a matter of fact, incentive measures, encompassing coupons and economic subsidies, have been extensively deployed in pro-environmental domains, including electric vehicles [18] and the use of disposable bags [19], to guide residents' decision-making behaviors. However, the impact of incentive measures on residents' intention to adopt low-carbon travel remains elusive. Zhang, Hu [20] assert that incentives do not significantly reinforce the intentional behavioral relationship concerning waste sorting, while Jenn, Springel [21] point that incentives indeed exert a substantial effect on the purchase intention of new energy vehicles. Thus, the intricate relationship between view of incentive and the low-carbon travel intention merits further exploration, as it bears potential impact for policy implementation.

Furthermore, people's behavioral intention is inherently influenced by socio-economic attributes. Although Boldero [22] contends that studies centering on psychological constructs possess greater explanatory power than those focusing on socio-economic attributes in predicting pro-environmental behavior, the divergences in socioeconomic attributes still warrant attention. Research by Li, Lo [23] discussed the influence of socio-economic characteristics on low-carbon travel behavior suggesting strong relationship between mode choice, gender, and monthly income. Besides, the impact of socioeconomic attributes on pro-environmental domain also been proved by Liu, Du [24] and Yang, Li [25] which suggest individuals with different socio-economic attributes has various preference in behavioral intention.

According the above literature, individuals' low-carbon travel behavior is strongly associated with psychological variables, policy interventions, and socioeconomic attributes. Therefore, this study not only integrates the TPB and VBN to construct a robust theoretical framework of low-carbon travel intention from a psychological perspective, but also introduces the policy variables and socio-economic attributes, which comprehensively analyzes the factors affecting low-carbon travel and the acceptance of the “carbon incentive” policy in mobility field.

The subsequent sections of this paper are organized as follows: Section 2 describes the theoretical framework and defines the interrelationships between the variables. In Section 3, the research methodology is explicated. The results and discussions section delve into an exposition of the factors exerting influence on low-carbon travel intention. Finally, the last section presents the key conclusions and recommendations for advancing low-carbon travel intention.

## 2 Theoretical framework

Pro-environmental behavior epitomizes a diverse spectrum of actions devoted to the preservation and mitigation of impacts on the natural environment [26]. TPB and VBN stand as foundational pillars, illuminating the intricacies of the interplay between human behavior and the environment. Low-carbon travel emerges as a prime example of pro-environmental behavior, capturing the interest of scholars and receiving wide attention.

### 2.1 The theory of planned behavior

TPB originally proposed by Ajzen [2], designed to illuminate the change of human behavioral patterns. Central to this theory is the construct behavioral intention, exerts impact on determining the decision of action individuals undertake or not. Thus, to unravel the mechanism of human behavioral decisions, a prerequisite undertaking involves delving into behavioral intention. This triad of constructs, namely attitude (AT), subjective norms (SN), and perceived behavioral control (PBC), serves as a profound and illuminating elements through which behavioral intention can be explicated.

In recent times, scholars have integrated TPB into low-carbon travel intention research, exploring the causal connections among these constructs [27,28]. There are also some researchers extracted and extended part of the constructs, such as subjective norms, to separately analyze their effects on energy-saving behaviors [29]. Within this study, attitude serves as people's perceptions of low-carbon travel, encompassing elements such as convenience. Subjective norms offer a reflection of social atmosphere and interaction surrounding low-carbon travel, while perceived behavioral control unveils individuals' cognitions of the challenges entailed in adopting low-carbon travel behavior. By and large, scholars hold that a positive attitude, subjective norms, and diminished perceived behavioral control shall promote behavioral intention. Nonetheless, Liu's work [24] points that attitude, subjective norms, and perceived behavioral control are not independent but are somewhat correlated, with both subjective norms and perceived behavioral control exerting their influence on low-carbon travel intention through their impact on attitude. Drawing on this empirical foundation, the subsequent hypotheses were formulated:

H1 Subjective norms positively impacts attitude.

H2 Perceived behavioral control positively impacts attitude.

H3 Attitude positively impacts behavioral intention.

H4 Perceived behavioral control positively impacts behavioral intention.

H5 Subjective norms positively impacts behavioral intention.

### 2.2 The value-belief-norm theory

VBN, a fusion of values, beliefs, and norms, was initially introduced by Stern [30] to unravel public support in environmental conservation. Undoubtedly, public support stands as a determinant in tackling social challenges like low-carbon travel, prompting numerous scholars to embrace VBN as an elemental foundation to research pro-environmental behavior [7].

The beliefs segment, as the underlying theory of this study, encompassing three constructs. The first construct is the new ecological environment paradigm (NEP), asserting an inseparable bond between human endeavors and the fragility of the biosphere. Initially postulated by Dunlap and Van Liere [31], the NEP further refined and validated by researchers as the evolution of social dynamics [32]. In this study, a subset of questions sourced from the NEP scale served as a litmus test to discern individuals' cognitive grasp of pro-environmental facets, referred to as low-carbon knowledge (LCK). Under the influence of NEP, the second belief construct, awareness of consequence (AC), takes shape. Embodied in pro-environmental behavior's altruistic essence, awareness of consequence reflects an individual's cognition of potential threats to others, resulting responsive actions to alleviate or eliminate such concerns. This phenomenon is encapsulated by the construct of ascription of responsibility (AR) [30]. Numerous researches demonstrated to the applicability of this theory in pro-environmental issues, and the interconnections of the VBN constructs can be explicated as follows: low-carbon knowledge - > awareness of consequence - > ascription of responsibility, thus laying the foundation for the ensuing hypothesis:

H6 Low-carbon knowledge positively impacts awareness of consequence.

H7 Awareness of consequence positively impacts ascription of responsibility.

H8 Awareness of consequence positively impacts behavioral intention.

H9 Ascription of responsibility positively impacts behavioral intention.

### 2.3 A merging theoretical framework

The widespread adoption of TPB and VBN in the domain of pro-environmental research substantiated the efficacy of these theories. However, TPB reflects rational behavior choices, whereas VBN stands for altruism. Recognizing the potential for enhanced explanatory potency, Bamberg and Möser [10] pointed the fusion of these two theories, thereby promoting scholars' pursuit of a combined TPB and VBN model. Consequently, Liu, Du [24] yielded an integrated intentional influence model, which offered insights into individuals' intention toward low-carbon travel. Nonetheless, that continues to draw scholars' attention to the intricate interplay between constructs derived from these theories [33]. Generally speaking, strong social pressure can lead to a certain ascription of responsibility, especially in the Chinese context where most people have a strong sense of face, and the corresponding improvement in the ascription of responsibility may affect people's attitude toward a specific behavior.

Moreover, in order to promote low-carbon travel to protect the environment, the Chinese government has made a lot of efforts, the prominent urbans (e.g. Beijing, Guangzhou) have started to implement low-carbon travel incentives, offering attractive rewards to motivate public engagement in eco-friendly behaviors which may change the perceived difficulty and social atmosphere towards low-carbon travel even improve the perceived responsibility of people. This approach mirrors similar incentive measurements applied to encourage the purchase of new energy vehicles. Nevertheless, the efficacy of incentives has resulted debates across diverse studies [20,21,34]. As such, incorporating the public's attitudes toward incentive, view of incentive (VI), within the model framework holds the promise of illuminating the impact of incentive implementation on low-carbon travel behavior. Drawing upon these deliberations, we propose the following hypotheses:

H10 Subjective norms positively impacts ascription of responsibility.

H11 Ascription of responsibility positively impacts attitude.

H12 View of incentive positively impacts attitude.

H13 View of incentive positively impacts subjective norms.

H14 View of incentive positively impacts perceived behavioral control.

H15 View of incentive positively impacts ascription of responsibility.

H16 View of incentive positively impacts behavioral intention.

**Fig. 1:**
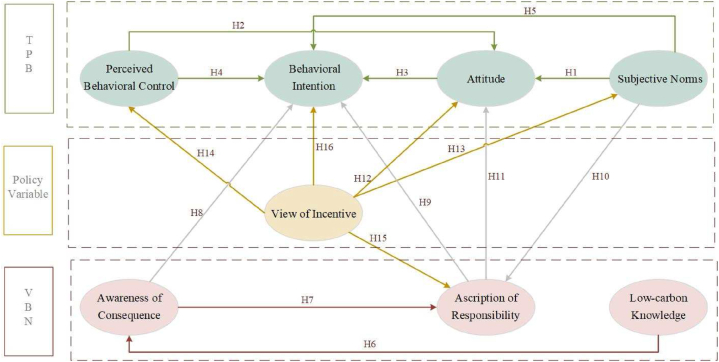
The theoretical framework of low-carbon travel intention.

The study introduces a comprehensive theoretical framework, comprising 8 constructs and 14 hypotheses. The influence relationships among these constructs is depicted in Fig. 1.

## 3 Research methodology

### 3.1 Ethical approval

This study was conducted according to the guidelines of the Declaration of Helsinki and approved by the Ethics Committee of college of biomedical engineering and instrument science, Zhejiang University, with ethics approval reference [2023]-71. The survey is conducted online, and there is no need to obtain respondents' real names or other information. Prior to conducting the survey, respondents will be briefed on the content of the survey, informed of the purpose of the data, and asked if they wish to take the survey. Therefore, respondents who take the survey are considered to have signed an oral informed consent form.

### 3.2 Measurement instruments

The measurement scale employed in this study were adopted to gather data and test the aforementioned hypotheses based on previously validated research. The questionnaire comprised two distinctive sections: (1) Individual Information, where an investigation of social-economic attributes, including gender, age, and household size, was conducted. (2) Construct Scale, instruments of TPB and VBN were applied in a broad range of disciplines [4,11,20,35]. The utilization of these well-validated scales elevates the questionnaire's reliability and validity, providing a sturdy basis for comparative analyses with other research. Moreover, we integrated the construct view of incentive to analyze the impact of incentive policies on low-carbon travel intention. Notably, the language employed in this survey underwent refinement, catering to the target sampling and fostering understanding of the questions, consequently minimizing potential measurement errors. To measure the degree of agreement/disagreement regarding indicators for all latent variables in this study, we employed a 5-point Likert scale [36], a widely embraced measurement approach, encompassing the range from 1 (disagree) to 5 (agree). The construct scale used in this paper is shown in Table 1.

**Table 1:** Construct scale.

Construct	Item	Issue
Attitude	AT1	I think low-carbon travel is safe
	AT2	I think low-carbon travel is affordable
	AT3	I think low-carbon travel is convenient
Subjective Norms	SN1	My friends or colleagues encourage me to use low-carbon travel modes
	SN2	My friends or colleagues often choose low-carbon travel modes
	SN3	My family often chooses low-carbon travel modes
Perceived Behavioral Control	PBC1	I don't think it's difficult to implement low-carbon travel behavior
	PBC2	Implementing low-carbon travel behavior hardly takes up my time
Ascription of Responsibility	AR1	Governments and businesses have a responsibility to protect the environment and reduce air pollution
	AR2	No matter what others do to travel, environmental values and responsibility guide me to travel in a low-carbon way
Awareness of Consequence	AC1	Low carbon travel can reduce pollution
	AC2	Low carbon travel can save energy
	AC3	Low-carbon travel can ease urban traffic congestion
	AC4	Low carbon travel can protect the environment
Low-carbon Knowledge	LCK1	Using new energy vehicles to travel can save energy and reduce emissions
	LCK2	Sudden acceleration or deceleration of the vehicle will increase energy consumption
	LCK3	Global warming will increase the frequency and intensity of extreme weather
	LCK4	Using public transportation instead of driving can reduce carbon emissions
View of Incentive	VI1	If there is a low-carbon travel incentive, I would like to give priority to low-carbon travel for my future daily trips
	VI2	I think the low carbon travel incentive will encourage me to travel in a low carbon way in the future
	VI3	I think low carbon travel incentives are necessary to encourage individuals travel in a low-carbon way
Behavioral Intention	BI1	I have a strong intention to use low-carbon travel modes in my daily travels
	BI2	I would like to use low-carbon travel modes in my daily travel in the future
	BI3	I would like to recommend others to choose low-carbon travel modes in the future

In pursuit of enhancing the questionnaire's precision in measurement, a pre-test was conducted prior to its finalization. Drawing insights from the pre-test revelations, subtle refinements were introduced, encompassing the calibration of language, expression, and the sequential arrangement of survey indicators. Furthermore, an introductory segment was integrated, aiming to illuminate the survey's objectives from the very outset. Additionally, to gauge the participants' attentive engagement, supplementary queries were included into the survey, inviting valuable reflections throughout the response process.

### 3.3 Study site and data collection

The survey was undertaken in Hangzhou, the capital of Zhejiang Province, China, during the period from February to March 2023. Total of 602 valid responses were gathered in collaboration with a reputable survey company boasting a vast user base of over 6 million registered individuals in China.

To ensure a portrayal of the population, probability proportional to size sampling method proposed by Skinner [37] was used. The distribution of gender, education, and age within the sample consistent with the percentages recorded in the 2021 demographic yearbook of Hangzhou, as shown in Table 2. And the distribution of respondents across each district within Hangzhou mirrored the predetermined proportions.

**Table 2:** General characteristics of respondents (N = 602).

Characteristics	Category	Frequency	Percentage of the sample	Percentage of demographic yearbook
Gender	Male	308	51.16%	52.08%
	Female	294	48.84%	47.92%
Age	<60	489	81.23%	80.61%
	≥60	113	18.77%	19.39%
Education	Junior high school and below (Low)	281	46.68%	52.14%
	Senior high school (Middle)	102	16.94%	16.46%
	University or above (High)	219	36.38%	31.40%
Residential area	West Lake District	79	13.12%	12.60%
	Yuhang District	128	21.26%	20.92%
	Gongsu District	98	16.28%	16.56%
	Binjiang District	35	5.81%	5.72%
	Qiantang District	79	13.12%	12.84%
	Xiaoshan District	92	15.28%	16.85%
	Linping District	36	5.98%	6.35%
	Shangcheng District	55	9.14%	8.15%
Household monthly income	Low income (<9999RMB^1^)	104	17.28%	–
	Middle income (10000RMB-19999RMB)	300	49.83%	–
	High income (20000RMB or above)	198	32.89%	–
Marriage	Yes	574	95.35%	–
	No	55	9.14%	–
Driver license	Yes	466	77.41%	–
	No	136	22.59%	–
Number of cars	0	126	20.93%	–
	1 unit	438	72.76%	–
	2 or more	38	6.31%	–
Children ownership	No	321	53.32%	–
	Yes	281	46.67%	–
Number of non-motor vehicles	0	57	9.47%	–
	1Unit	251	41.69%	–
	2 or more	294	48.84%	–
Household structure	All adult family	207	34.39%	–
	Family with children	281	46.68%	–
	Elder family	114	18.94%	–

Note：- implies no relevant data in the demographic yearbook.

1 RMB is the monetary unit of China.

**Fig. 2:**
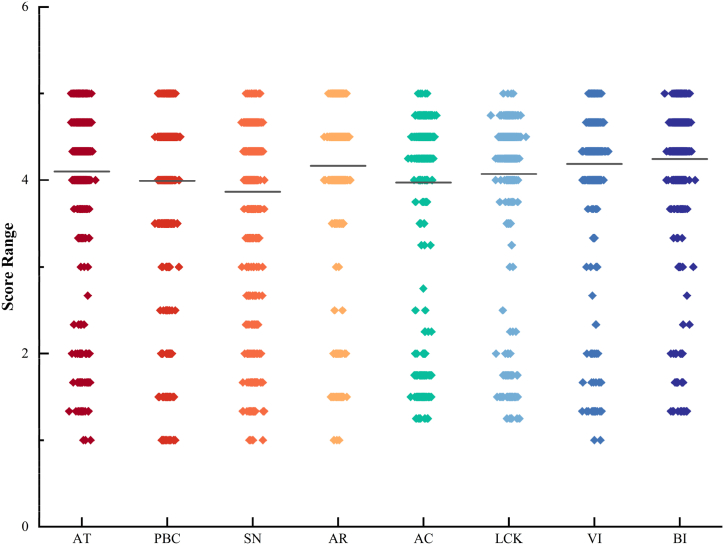
Score of constructs.

In the second section of the questionnaire, eight constructs, namely attitude, perceived behavioral control, subjective norms, ascription of responsibility, awareness of consequence, low-carbon knowledge, view of incentive, and behavioral intention were investigated. For each construct, we computed the average scores of all indicators, and scores have been illustrated in Fig. 2. Remarkably, a prevailing preference toward low-carbon travel was discerned among the respondents, evident through the high scores across these constructs. This inclination can, in part, be ascribed to the potent impact of government initiatives, coupled with the ever-increasing ease and accessibility of low-carbon travel alternatives. The high scores achieved in the view of incentive construct signify high allure of incentive, likely to entice most individuals into low-carbon travel.

### 3.4 Modeling procedure and tools

To construct the Structural Equation Model (SEM), we employed a two-stage approach, following the proposal by Anderson and Gerbing [38]. In the first stage, we conducted confirmatory factor analysis (CFA) to evaluate the sample by reliability and validity. Subsequently, the establishment of the SEM enabled us to explore the hypotheses, relying on the path coefficients. Multi-group method was used to analyze the heterogeneity of individuals with diverse socio-economic attributes according the SEM. In this approach, SPSS version 26 and AMOS version 21 software were used in combination, which was widely applied for its user-friendliness [18,39].

## 4 Results and discussions

### 4.1 Confirmatory factor analysis

To ensure the reliability and validity of the sample, CFA was adopted by maximum likelihood estimation as the first step of the two-stage approach, which requires to construct a factor analysis model assumes that all the latent variables are independent.

In order to evaluate the model fitness, sample size-independent indices were selected, such as goodness of fit index (GFI), adjusted goodness of fit index (AGFI), chi-square/degrees of freedom (Chi/df), and robustness of mean squared error approximation (RMSEA), which is also known as structural validity. Remarkably, the Chi/df value of 2.042 fell below the acceptable threshold of 3, while both the GFI score of 0.937 and the AGFI score of 0.91 exceeded the recommended benchmark of 0.9 [40,41]. Moreover, the RMSEA value of 0.042 was below the threshold of 0.08, collectively suggesting the satisfactory fit. Additionally, we evaluated the reliability, convergent validity, and discriminant validity of all latent variables.

**Fig. 3:**
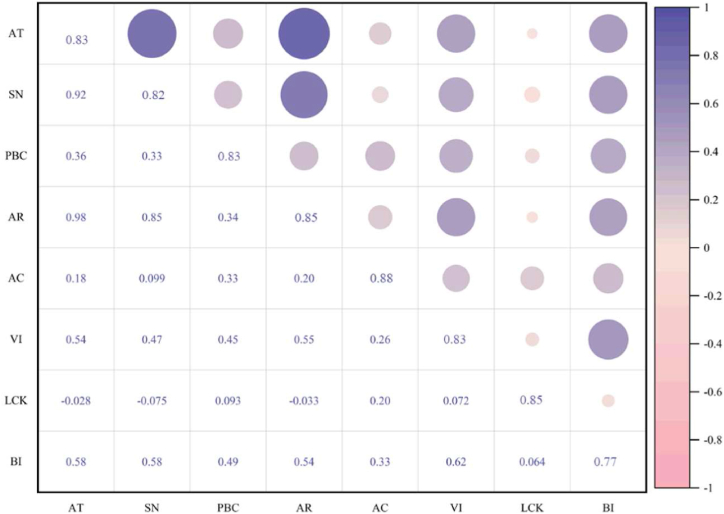
Test of discriminant validity.

Cronbach's Alpha was employed to verify the internal consistency of items within each construct. As shown in Table 3, Cronbach's Alpha surpassed the threshold of 0.7, exhibiting values ranging from 0.81 to 0.926, indicative of internal consistency [42]. Convergent validity, the hallmark of theoretical interconnections among items within a construct, was assessed by factor loading, composite reliability (CR) and average variance extracted (AVE). In this study, factor loadings ranged from 0.75 to 0.909, while AVE values spanned between 0.596 and 0.695, surpassing the minimum benchmark of 0.5 [43], CR ranged from 0.813 to 0.935 surpassing the threshold of 0.7. Crucial for distinguishing between constructs, discriminant validity quantifies the independence among them. Fig. 3 displays the results, revealing that most correlation coefficients between constructs are lower than the squared AVE values along the diagonal, underscoring the robustness of discriminant validity [43].

**Table 3:** Test of reliability and convergent validity.

Latent variable	Item	Factor loading	Cronbach's Alpha	CR	AVE
AT	AT1	0.837	0.872	0.872	0.695
	AT2	0.846			
	AT3	0.818			
SN	SN1	0.794	0.881	0.859	0.671
	SN2	0.776			
	SN3	0.883			
PBC	PBC1	0.869	0.81	0.813	0.686
	PBC2	0.785			
AR	AR1	0.876	0.881	0.883	0.715
	AR2	0.802			
	AR3	0.857			
AC	AC1	0.892	0.926	0.935	0.782
	AC2	0.826			
	AC3	0.908			
	AC4	0.909			
VI	VI1	0.838	0.866	0.870	0.691
	VI2	0.856			
	VI3	0.799			
LCK	LCK1	0.856	0.905	0.914	0.723
	LCK2	0.813			
	LCK3	0.841			
	LCK4	0.9			
BI	BI1	0.75	0.811	0.816	0.596
	BI2	0.739			
	BI3	0.825

### 4.2 Structural equation modeling

#### 4.2.1 Model fitting and modification

In the second phase of our two-stage methodology, we undertook an examination of the hypotheses proposed in this study. Upon executing the SEM model, Table 4 disclosed the fitness of hypothesis model, with a Chi/df value of 2.221, a GFI score of 0.932, an AGFI score of 0.912, and an RMSEA of 0.045. By removing the rejected hypotheses, hypothesis model was modified. However, according to the theory of planned behavior, attitude and subjective norms usually have a significant effect on behavioral intention. Removing these two paths simultaneously would undermine the theoretical foundation of the model. In the past studies [4], the effect of attitude on behavioral intention is usually higher than the effect of subjective norms, and the study of Liu, Du [24] pointed subjective norms not yield a significant impact on low-carbon travel intention in Tianjin, China, which is a city with a similar cultural background and city size to Hangzhou in the study area. Therefore, we retained the path from attitude to behavioral intention to maintain the integrity of the model theory for final model. And the modification indices derived from AMOS suggested potential enhancement in the model. Specifically, it proposed the addition of two supplementary paths: one from the view of incentive to awareness of consequence, and the other from awareness of consequence to perceived behavioral control. The incorporation of these paths led to an enhancement in the model's fitness, culminating in a Chi/df value of 1.991, a GFI score of 0.937, an AGFI score of 0.919, and an RMSEA of 0.041, while the core theoretical underpinnings of this study remained undisturbed. Consequently, this refined version (called final model) was retained for further analysis.

**Table 4:** The results of the structural model fitness.

Model Fit Indices	Acceptable Value	Base Model	Hypothesis Model	Final Model
Chi/df	<3	2.342	2.221	1.991
GFI	>0.9	0.937	0.932	0.937
AGFI	>0.9	0.918	0.912	0.919
RMSEA	<0.08	0.047	0.045	0.041
R^2^ of BI	–	0.420	0.529	0.530

Additionally, we developed a fundamental model without the construct view of incentive (called base model) to facilitate a comparation, thereby delving into the influence of incentives on behavioral intention. The fitness results of the base model displayed a Chi/df value of 2.342, a GFI score of 0.937, an AGFI score of 0.918, and an RMSEA of 0.047.

The final model showed a superior performance with a Chi/df of 1.991 and an RMSEA of 0.041 in comparison to the base model and hypothesis model. Moreover, it exhibited enhanced predictive capabilities for low-carbon travel intention, as substantiated by an R^2^ of 0.53, surpassing the performance of the basic model (R^2^ = 0.421) and hypothesis model (R^2^ = 0.529).

#### 4.2.2 Analysis of psychological determinants

All the hypotheses between constructs proposed by this study were tested. The results of hypothesis model are shown in Table 5 which demonstrates that H2, H3, H5, H9 and H12 fails the test of significance and thus the hypotheses are rejected.

**Table 5:** The results of the hypothesis model.

	Path			Standardized Estimate	*P*-Value	Hypothesis
H1	SN	→	AT	0.226	***	Supported
H2	PBC	→	AT	0.024	0.239	Rejected
H3	AT	→	BI	0.317	0.415	Rejected
H4	PBC	→	BI	0.11	***	Supported
H5	SN	→	BI	0.143	0.159	Rejected
H6	LCK	→	AC	0.227	***	Supported
H7	AC	→	AR	0.065	0.021	Supported
H8	AC	→	BI	0.052	0.014	Supported
H9	AR	→	BI	−0.26	0.384	Rejected
H10	SN	→	AR	0.686	***	Supported
H11	AR	→	AT	0.709	***	Supported
H12	VI	→	AT	−0.01	0.783	Rejected
H13	VI	→	SN	0.603	***	Supported
H14	VI	→	PBC	0.584	***	Supported
H15	VI	→	AR	0.188	***	Supported
H16	VI	→	BI	0.285	***	Supported

Note: *** stands for the *P*-value<0.001 and the same meaning as below.

**Fig. 4:**
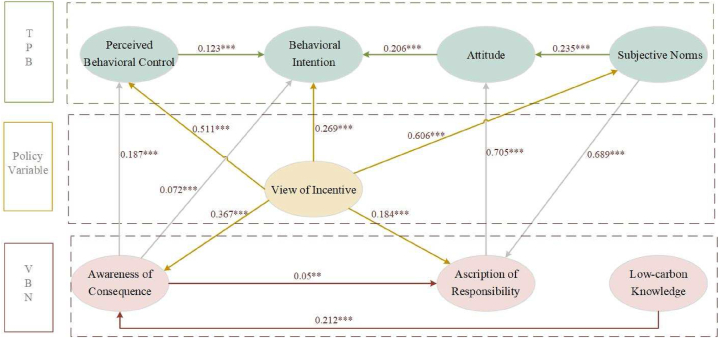
Final model framework.

The framework of final model is shown in Fig. 4 and the details are shown in Table 6. Note that H3 is supported in the final model, which seems conflicts with its rejection in the hypothesis model, possibly due to a degree of covariance resulting from the high correlation between attitude and subjective norms in the hypothesized model. Similar attempts were made for the remaining removed hypotheses, but no other significant paths were found indicating the reliability of supporting H3.

**Table 6:** The results of the final model.

Path				Standardized Estimate	*P*-Value	Hypothesis
H1	SN	→	AT	0.235	***	Supported
H3	AT	→	BI	0.288	***	Supported
H4	PBC	→	BI	0.206	***	Supported
H6	LCK	→	AC	0.212	***	Supported
H7	AC	→	AR	0.05	0.018	Supported
H8	AC	→	BI	0.072	***	Supported
H10	SN	→	AR	0.689	***	Supported
H11	AR	→	AT	0.705	***	Supported
H13	VI	→	SN	0.606	***	Supported
H14	VI	→	PBC	0.511	***	Supported
H15	VI	→	AR	0.184	***	Supported
H16	VI	→	BI	0.206	***	Supported
F1	VI	→	AC	0.367	***	Found
F2	AC	→	PBC	0.187	***	Found

The revelations unveiled by Table 5, Table 6 illuminate the interrelationships among the constructs. As for the constructs from TPB, the impact of subjective norms on attitude emerges as a significant influence, while the effect of perceived behavioral control on attitude remains insignificant. Interestingly, both attitude and perceived behavioral control manifest a positive influence on behavioral intention as the evidence found in prior scholarly works [4,24]. As such, hypotheses H1, H3, and H4 were considered acceptable, while H2, H5 were rejected. Subsequently, the hypotheses around the framework of VBN were explored, which indicated H6, H7, and H8 were supported, while H9 was rejected. This indicated that the ascription of responsibility may not influence the low-carbon travel intention. Moreover, we also verified the interplay between TPB and VBN. Ascription of responsibility was influenced by subjective norms, exerting a simultaneous influence on attitude, which suggests the H10, and H11 were supported demonstrating the interaction of TPB and VBN. Guided by the modification indices, a new path F2 from awareness of consequence to perceived behavioral control was found. This discovery emphasizes the implications of consequence awareness on shaping the perceived difficulty of low-carbon travel.

Although many researchers have explored the intention towards low-carbon travel through diverse psychological theories, only a few unravel the effect of the view of incentive on it. As demonstrated in Table 6, the view of incentive exerts a positive influence on behavioral intention, and it also has a significant indirect impact on behavioral intention through subjective norms and perceived behavioral control. Furthermore, the modification indices suggest an additional path on awareness of consequence (F1). However, the influence on attitude seems insignificant, resulting in the validation of H13-H16, while H12 was rejected.

#### 4.2.3 Analysis and comparation of view of incentive

Upon the confirmed hypotheses, Table 7 and Table 8 show the total effects of each construct on low-carbon behavioral intention for base model and final model, respectively. Without considering view of incentive, attitude play a key role in low-carbon travel intention, followed by subjective norms. Overall, the influence of the constructs in the TPB is higher than that of the constructs in the VBN. The relative importance of the factors influencing low-carbon intentions was ranked as follows: attitude (AT), subjective norms (SN), ascription of responsibility (AR), awareness of consequence (AC), perceived behavioral control (PBC), and low-carbon knowledge (LCK). Intriguingly, although previous studies have argued that low-carbon knowledge is critical to low-carbon travel intentions [15,25], our investigation suggests that its influence is limited. This divergence might cause by the widespread dissemination of low-carbon knowledge, resulting in its less pronounced impact on travel decisions.

**Table 7:** Standardized coefficients for the effects of constructs on behavioral intention (Base Model).

Path			Direct Effect	Indirect Effect	Total Effect	Rank
AT	--->	BI	0.471	–	0.471	1
PBC	--->	BI	0.322	–	0.322	5
SN	--->	BI	–	0.426	0.426	2
AR	--->	BI	–	0.347	0.347	3
AC	--->	BI	0.18	0.146	0.326	4
LCK	--->	BI	–	0.063	0.063	6

**Table 8:** Standardized coefficients for the effects of constructs on behavioral intention (Final Model).

Path			Direct Effect	Indirect Effect	Total Effect	Rank
AT	--->	BI	0.288	–	0.288	2
PBC	--->	BI	0.205	–	0.205	5
SN	--->	BI	–	0.243	0.243	3
AR	--->	BI	–	0.213	0.213	4
AC	--->	BI	0.139	0.059	0.199	6
LCK	--->	BI	–	0.036	0.036	7
VI	--->	BI	0.351	0.283	0.634	1

Take view of incentive into model, the influence produced by view of incentive far exceeds that of attitude as the most important influence factor, which indicates that view of incentive emerges as the preeminent driving force, underscoring its pivotal role in fostering low-carbon travel. In other words, the implementation of low-carbon incentive policies has the potential to increase the enthusiasm for low-carbon travel. It is worth noting that under the high influence of view of incentive, the relative importance ranking among constructs influencing low-carbon travel intention has not changed much, and there is only an adjustment in the two of perceived behavioral control and awareness of consequence, but the influence effect of each constructs has been reduced, that is to say, the influence of low-carbon travel incentives on people's low-carbon travel intention exceeds the travelers' assessment of inherent attributes of low-carbon travel, such as convenience. Carbon incentives will work well to encourage low-carbon travel.

### 4.3 Heterogeneity analysis on socio-demographic attributes

In order to distinguish the heterogeneity inherent within the population, we adopted a multi-group approach to analyze the moderating effect which has been applied in a few existing literature [4]. Drawing upon the socio-demographic characteristics presented in Table 2, we selected five attributes, gender, education, household structure, household monthly income, and children ownership to stratify the survey sample. In the estimation process for each distinctive group, certain paths were revealed to be insignificant with specific categories. Consequently, the path from low-carbon knowledge to awareness of consequence was deemed unsuitable for the education group and accordingly been excluded. Similarly, the path from ascription of responsibility to attitude was found to be inapplicable to the elder family group which also been removed. Thus, the significant coefficients of influence paths among constructs, tailored for groups with diverse socio-demographic attributes is shown in Table 9.

**Table 9:** The results of the multi-group analysis.

Path				Gender		Education			Household Structure			Household Monthly Income		Children Ownership
				Male	Female	Low	Mid	High	Adult Family	Family with Children	Elder Family	Middle	High	No
H1	SN	--->	AT	0.308***	0.243^a^	0.717^a^	0.15^a^	0.263^a^	0.259^a^	0.303***	0.014***	0.373***	0.179^a^	0.23^a^
H3	AT	--->	BI	0.282***	0.296***	0.192^a^	–	0.674***	0.285***	0.275***	0.374^a^	0.336***	0.286***	0.279***
H4	PBC	--->	BI	0.237***	0.184^b^	–	0.413^a^	–	0.151^c^	0.327***	–	–	0.325***	–
H6	LCK	--->	AC	0.178^a^	0.181^a^	–	–	–	0.375***	–	0.51***	0.374***	–	0.399***
H7	AC	--->	AR	0.095^a^	–	–	0.106***	0.09^a^	0.086^c^	0.076^c^	–	0.101^a^	–	–
H8	AC	--->	BI	0.143^a^	0.139^b^	0.201^a^	0.363^a^	–	0.126^c^	0.155^a^	–	–	0.182^a^	0.125^a^
H10	SN	--->	AR	0.781***	0.754***	0.928***	0.639***	0.742***	0.81***	0.743***	0.96***	0.689***	0.851***	0.79***
H11	AR	--->	AT	0.714***	0.761***	–	0.888***	0.738***	0.766***	0.7***	–	0.669***	0.814***	0.795***
H13	VI	--->	SN	0.515***	0.444***	0.443***	0.283^a^	0.66***	0.351***	0.562***	0.647***	0.371***	0.505***	0.382***
H14	VI	--->	PBC	0.446***	0.358***	0.206^a^	–	0.702***	0.363***	0.5***	–	0.358***	0.395***	0.3***
H15	VI	--->	AR	0.148^a^	0.185***	–	0.24^a^	0.216***	0.143^a^	0.164^a^	–	0.219***	0.088^c^	0.173***
H16	VI	--->	BI	0.361***	0.324***	0.359***	0.255^a^	0.316***	0.379***	0.285***	0.322^a^	0.382***	0.282***	0.413***
F1	VI	--->	AC	0.246***	0.241***	0.285***	0.372***	0.317***	0.227***	0.243***	0.332^a^	–	0.295***	0.236***
F2	AC	--->	PBC	0.149^b^	0.279***	0.404***	0.301^a^	–	0.301***	0.124^a^	0.353^a^	0.275***	0.168^a^	0.323***

Note: *** stands for the *P*-value<0.001.

a stands for the *P*-value <0.01.

b stands for the *P*-value <0.05.

c stands for the *P*-value <0.1.

The hypotheses (H1, H3, H4, H6, H7) derived from TPB and VBN were not supported across the diverse cohorts. Subjective norms exert a more substantial impact on males’ attitudes (0.308 > 0.243) in contrast to female, presenting a result inconsistent with prior literature [44]. However, Liu, Du [24] pointed the gender disparity in driving behavior, with fewer female drivers in China, resulting in a greater reliance on public transit for females. This heavy reliance on public transit may made an insensitivity to subjective norms. Similarly, elder families displayed a diminished susceptibility to subjective norms when compared to other family types. Nonetheless, within these families, the influence of attitude on behavioral intention was most pronounced. Additionally, the behavioral intention of individuals with higher education were notably influenced by their attitudes than other groups. In our study, attitude primarily reflected considerations of the convenience and safety of low-carbon travel, suggesting that elders and individuals with higher education may accord paramount importance to convenience and safety while formulating their travel plans. Concerning the impact of perceived behavioral control on behavioral intention, significant disparities were unveiled based on household structure, with families with children evincing the highest degree of sensitivity. This implies that individuals residing with children may be more conscientious of perceived travel challenges, likely stemming from considerations of caregiving responsibilities. Furthermore, disparities were evident in the effect of awareness of consequence on behavioral intention. Those with higher education exhibited heightened awareness of the environmental benefits of low-carbon travel, consequently making a more profound impact on their behavioral intention.

The exploration of the interaction of psychological determinants (H8, H10, F2) uncovered some heterogeneity. Notably, individuals with limited education or elders displayed a conspicuous susceptibility to subjective norms, impacting their awareness of consequence. Such individuals may possess a somewhat diminished cognizance of the environmental benefits inherent in low carbon travel. Consequently, impacted by figures who important to them (called subjective norms), they tend to accept the concept of low-carbon travel as a means of environmental preservation. In addition, the physical constraints faced by elders, often coupled with constrained financial resources, may serve as an impetus for their adoption of low-carbon travel.

The hypotheses concerning the view of incentive (H13, H14, H15, H16, F1) evince significant and positive impact across all the diverse groups, thereby indicating the importance of incentives in fostering low-carbon travel. Nevertheless, the impact manifested variations among the distinct groups. Specifically, males exhibited heightened susceptibility to the influence of the view of incentive, this may be similar to the effect of subjective norms, owing to the prevalent use of public transit among females and the greater reliance on car travel among males, the promotion of low-carbon travel intentions among females may be comparatively less potent in response to incentives. Furthermore, families with children demonstrated a more pronounced impact on subjective norms and perceived behavioral control, in contrast to childless families, plausibly attributed to the responsibilities entailed in caring for children. Given that individuals in these families tend to opt cars in their daily routines, fostering a favorable view of incentives can elevate the overall climate of low-carbon travel while concurrently alleviating the perceived challenges associated. It is worth noting that despite the impact on subjective norms and perceived behavioral control, the effect on behavioral intention remains somewhat weak, which aligns with intuitive reasoning. In addition, elder families also exhibited a notable susceptibility to the influence of the view of incentive. The presence of significant pathways related to the view of incentive across diverse socio-economic groups manifest the effectiveness of incentives in enhancing the intention towards low-carbon travel.

## 5 Conclusions and policy implications

Drawing from TPB and VBN, this study is devoted to exploring the theoretical framework of low-carbon travel intention and analyzing population heterogeneity with diverse socio-economic attributes. Meanwhile, it also aims to verify the feasibility of incentive policy in fostering low-carbon travel.

Based on the results of SEM, psychological latent variables from TPB and VBN are all found significant impact on low-carbon behavioral intention. Notably, only part of them have direct impact on it. In terms of the total effect, variables from TPB have more strong impact than those from VBN, suggesting attitude and subjective norms are the most important determinants. Low-carbon knowledge, which was seen as an essential influencing factor in pro-environmental domain [4,24,35], only has a limited influence on low-carbon travel behavioral intention. View of incentive, as a construct introduced in the theoretical framework, has increased the explanatory power of SEM which raised R^2^ of behavioral from 0.421 to 0.53. And the total effect of view of incentive exerted on behavioral intention is the strongest among all the latent variables, similar phenomenon was found on research proposed by Li, Zhao [27] demonstrating the impact of policy is salient. The strong effect of view of incentive manifest the high attraction on promoting low-carbon travel intention. In light of the diversity in sociodemographic attributes among individuals, population heterogeneity was revealed by multi-group analysis. Male show susceptibility to both subjective norms and the view of incentive. Similarly, elder households, constituting a distinctive cohort, also exhibit heightened responsiveness to view of incentive. The existence of children yields a significant impact, wherein the view of incentive exercises a significant impact on subjective norms and perceived behavioral control for individuals with young ones. Nonetheless, the positive effect on low-carbon travel intention remains relatively modest.

According the above findings, the following policy implications are proposed in this paper. Firstly, the impact of view of incentive confirm the applicability of incentive measurement, however, individuals with different sociodemographic attributes have varying susceptibility to incentives. Since the sociodemographic attributes such as gender are immutable, tailored incentive measurements may be needed to cultivate a thriving inclination towards low-carbon travel. Hence, we urge policymakers to design robust regulatory frameworks for the carbon trading market which will be bedrock of the incentive measurement implementation (e.g. carbon credits). Secondly, since the high impact of children on behavioral intention, policymakers must holistically address the specific requirements of individuals with children, concurrently improving the safety and convenience of low-carbon travel infrastructures while introducing family-friendly travel policies (e.g., specialized seats for children) to inspire a transformative shift towards low-carbon travel. Lastly, attitude and subjective norms are as key influencing factors for low-carbon behavioral intention, and low-carbon knowledge only has limited impact, efforts to expand public transportation services area and the dissemination of persuasive propaganda about low-carbon travel are both vital in positively changing the attitude toward low-carbon travel and increasing the perception of social pressure, and finally nurturing a positive atmosphere for low-carbon travel. While the promotion of low-carbon knowledge be seen as a supplementary measure.

In summation, this study proposes a theoretical framework, designed to illuminate low-carbon travel intention, which can be used to analyze the intention of residents in other regions with similar characteristics. The empirical validation stands for the feasibility of low-carbon incentive policies. And the population heterogeneity also been explored. However, there still exist some limitations. Firstly, despite the robustness inherent in the theoretical framework, certain factors, notably travel habits, warrant additional refinement to strengthen the accuracy and completeness of the model. Secondly, the integration of the TPB and VBN improves the degree of explanation of low-carbon travel intention, the intention and behavior are not perfectly aligned, and therefore the differences and impacts of inconsistencies between them need to be considered in the future. Thirdly, while the impact of the view of incentive on low-carbon intention is examined, the potential influence of incentive measures on actual low-carbon behavior remains an uncharted territory. Accordingly, the implementation of stated preference experiments is needed for future analysis.

## Funding

This work was supported by the “Pioneer” and “Leading Goose” R&D Program of Zhejiang (2023C01240); 10.13039/501100001809National Natural Science Foundation of China (Grant No. 52131202); the Center for Balance Architecture, 10.13039/501100004835Zhejiang University.

## Ethics statement

This study was reviewed and approved by the Ethics Committee of college of biomedical engineering and instrument science, Zhejiang University, with the approval number: [2023]-71.

## Data availability statement

Data will be made available on request.

## CRediT authorship contribution statement

**Yan He:** Writing – original draft, Methodology, Investigation, Formal analysis, Data curation. **Yilin Sun:** Writing – review & editing, Supervision, Investigation, Funding acquisition, Conceptualization. **Zhijian Zhao:** Writing – review & editing, Visualization, Investigation. **Mengwei Chen:** Writing – review & editing. **E. Owen D. Waygood:** Writing – review & editing. **Yang Shu:** Writing – review & editing.

## Declaration of competing interest

The authors declare that they have no known competing financial interests or personal relationships that could have appeared to influence the work reported in this paper.
